# Development of Genetic Tools for the Manipulation of the Planctomycetes

**DOI:** 10.3389/fmicb.2016.00914

**Published:** 2016-06-16

**Authors:** Elena Rivas-Marín, Inés Canosa, Eduardo Santero, Damien P. Devos

**Affiliations:** ^1^Laboratory of Evolutionary Innovations, Centro Andaluz de Biología del Desarrollo, Consejo Superior de Investigaciones Científicas, Universidad Pablo de OlavideSeville, Spain; ^2^Microbiology Area, Centro Andaluz de Biología del Desarrollo, Consejo Superior de Investigaciones Científicas, Universidad Pablo de OlavideSeville, Spain

**Keywords:** PVC superphylum, Planctomycetes, *Gemmata obscuriglobus*, *Gimesia maris*, *Planctopirus limnophila*, *Blastopirellula marina*, genetic tools, insertion mutant

## Abstract

Bacteria belonging to the Planctomycetes, Verrucomicrobia, Chlamydiae (PVC) superphylum are of interest for biotechnology, evolutionary cell biology, ecology, and human health. Some PVC species lack a number of typical bacterial features while others possess characteristics that are usually more associated to eukaryotes or archaea. For example, the Planctomycetes phylum is atypical for the absence of the FtsZ protein and for the presence of a developed endomembrane system. Studies of the cellular and molecular biology of these infrequent characteristics are currently limited due to the lack of genetic tools for most of the species. So far, genetic manipulation in Planctomycetes has been described in *Planctopirus limnophila* only. Here, we show a simple approach that allows mutagenesis by homologous recombination in three different planctomycetes species (*i.e.*, *Gemmata obscuriglobus*, *Gimesia maris*, and *Blastopirellula marina*), in addition to *P. limnophila*, thus extending the repertoire of genetically modifiable organisms in this superphylum. Although the Planctomycetes show high resistance to most antibiotics, we have used kanamycin resistance genes in *G. obscuriglobus*, *P. limnophila*, and *G. maris*, and tetracycline resistance genes in *B. marina*, as markers for mutant selection. In all cases, plasmids were introduced in the strains by mating or electroporation, and the genetic modification was verified by Southern Blotting analysis. In addition, we show that the green fluorescent protein (*gfp*) is expressed in all four backgrounds from an *Escherichia coli* promoter. The genetic manipulation achievement in four phylogenetically diverse planctomycetes will enable molecular studies in these strains, and opens the door to developing genetic approaches not only in other planctomycetes but also other species of the superphylum, such as the Lentisphaerae.

## Introduction

The PVC superphylum comprises the Planctomycetes, Verrucomicrobia, Chlamydiae (PVC) superphylum comprises the Planctomycetes, the Verrucomicrobia and the Chlamydiae phyla, but also several others, including the Lentisphaerae (Wagner and Horn, 2006; Fuerst, 2013). Bacteria belonging to the Planctomycetes present peculiar features that are rare in bacteria (Devos, 2013; Fuerst, 2013; Devos and Ward, 2014), and some of which are more frequent in archaea or eukaryotes (Devos and Reynaud, 2010; Reynaud and Devos, 2011). Typical examples are the lack of the protein FtsZ and the division mode by budding in some Planctomycetes, the synthesis of sterol (Pearson et al., 2003; Desmond and Gribaldo, 2009), or the presence of a complex endomembrane system (Devos, 2014). Also, the Planctomycetes represent an exceptional clade within the domain Bacteria because they are main players in the global nitrogen and carbon cycles (Strous et al., 1999; Lindsay et al., 2001; van Niftrik et al., 2004).

Despite the interest in the study of these and other peculiar features, their detailed characterization has so far been limited, mainly to computational genomes and proteomes analyses, and microscopy, due to the lack of genetic tools to manipulate the organisms.

Taking into account that those bacteria are fastidious, *i.e.*, their doubling times are in the order of a few hours (Jetten et al., 1998), they tend to aggregate into big clamps and they are naturally resistant to many antibiotics (Cayrou et al., 2010), it is not surprising that little is known about their genetic manipulation. So far, the only species that has been successfully genetically modified is *Planctopirus limnophila*, which shows one of the fastest growth rates among the cultured Planctomycetes (Jogler et al., 2011). Furthermore, endogenous plasmids and bacteriophage have been reported (Ward-Rainey et al., 1996; Ward et al., 2006; Labutti et al., 2010), and its genome sequence is available (Labutti et al., 2010). Genetic manipulation of *P. limnophila* by electroporation of circular or linear DNA, containing homologous sequence of the chromosome, has been achieved (Jogler et al., 2011; Erbilgin et al., 2014), and also by transposon mutagenesis using the EZ-Tn5 transposome (Epicenter) (Schreier et al., 2012).

**FIGURE 1:**
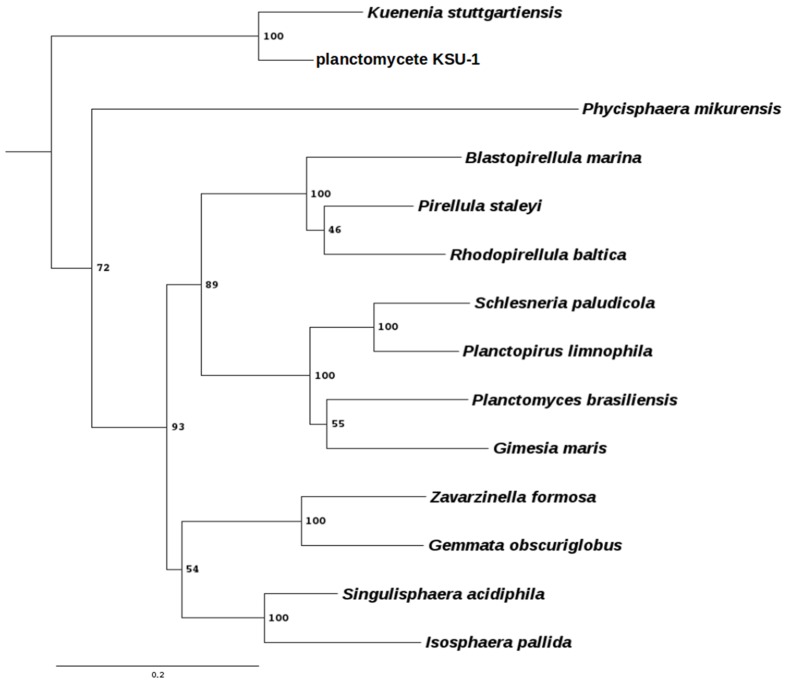
**Phylogenetic tree of some planctomycetes species.** Phylogenetic profiling was done using the RNA-polymerase subunit β protein, encoded by the *rpoB* gene. Protein sequences were extracted using BLAST (Altschul and Lipman, 1990) with an e-value of 1e^-5^. The multiple sequence alignment was done using Clustal Omega (Sievers et al., 2011) with default parameters and manually curated. The tree was generated using PhyML 3.1 (Guindon, 2010) using the LG matrix, 100 bootstraps, tree and leaves refinement, SPR moves and amino acids substitution rates were determined empirically. Bootstrap values are indicated at the nodes. Scale bar indicates the amount of substitution per site. The chlamydiae *Waddlia chondrophila* was used as an outgroup.

To expand our knowledge about these peculiar bacteria, we have developed new strategies for genetic manipulation of other members of this group. We have selected the three following strains; *Gemmata obscuriglobus* UQM 2246 (Franzmann and Skerman, 1984), *Blastopirellula marina* DSM 3645 (Schlesner et al., 2004), and *Gimesia maris* DSM 8797 (Bauld and Staley, 1976), as they belong to the three main branches of the Planctomycetes phylum (**Figure 1**). *G. obscuriglobus* shows so far, some of the most interesting features of this phylum, which are the presence of membrane coat-like proteins, one of which has been located in tight interaction with the membranes of the periplasmic vesicles (Santarella-Mellwig et al., 2010), its ability to internalize whole proteins prior to their intracellular degradation (Lonhienne et al., 2010), or the presence of sterol embedded in their membranes (Pearson et al., 2003). *G. maris* was selected because it is susceptible to DNA acquisition by biparental mating with distantly related strains such as the Gram negative *Pseudomonas putida* (Dahlberg et al., 1997, 1998). Finally, *B. marina*, a phylogenetically distant strain to *G. obscuriglobus, G. maris*, and *P. limnophila* within the Planctomycetes clade was selected in an attempt to cover the diversity of the Planctomycetes phylum as broadly as possible.

In the present study, we developed an efficient targeted-gene disruption approach by homologous recombination in three strains, which have never been genetically modified, as well as *P. limnophila*. The gene encoding for a beta lactamase (*bla* gene) has been selected for mutagenesis since multiple copies are found in the genomes and seems to be non-essential. Both interruption and deletion of the gene have been explored. This method represents an initial framework to develop new genetic tools in other Planctomycetes and opens up enormous fields of research amongst which the possibility to explore the cellular biology of those peculiar bacteria stands out.

## Materials and Methods

### Bacterial Strains and Culture Conditions

The bacterial strains used in this work are summarized in **Table 1**. *Escherichia coli* was grown in Luria–Bertani medium (LB) at 37°C, *G. obscuriglobus* UQM 2246 in LB NaCl-free at pH 7.2, *G. maris* DSM 8797 in Maris broth (C. Jogler, DSMZ, Braunschweig, Germany, personal communication), *B. marina* DSM 3645 in M14 medium (DSMZ medium 600. M14) and *P. limnophila* DSM 3776 in a modified PYGV medium (DSMZ medium^1^ 621: 0.1% yeast extract, 0.1% peptone, 0.1% glucose, 10 mM HEPES (pH 7,5), vitamin solution, and Hutners basal salt solution from DSMZ 590 medium). All planctomycetes were grown at 28°C. 1.5% bacto-agar was added for solid medium. To avoid contamination of the planctomycetes cultures, cycloheximide 50 μg ml^-1^ and ampicillin 100 μg ml^-1^ were added. Cultures were grown aerobically in a shaker (180 rpm) using 125 or 250 ml baffled flasks closed with aluminum stopper filled with 25 or 50 ml of medium, respectively (one fifth of the volume of the flask). When required, antibiotics were used at the following concentrations (μg ml^-1^): kanamycin (Km) 30 for *G. obscuriglobus*, 50 for *P. limnophila* and 60 for *G. maris* and, tetracycline (Tc) 2.5 for *B. marina*. All reagents were purchased from Sigma–Aldrich.

**Table 1:** Strains used in this study.

Strain name	Genotype	Reference
*Escherichia coli* DH5α	F^-^ϕ80*lacZ*ΔM15Δ(*lacZYA-argF)U169 recA1 endA1 hsdR17*(r_K_^-^m_K_^-^) *supE44 thi-1 gyrA relA1*	Hanahan, 1983
*Gemmata obscuriglobus* UQM 2246	Wild type (WT) strain	Franzmann and Skerman, 1984
*G. obscuriglobus* UQM 2246 DV001	*bla*’::pDV003	This work
*Gimesia maris* DSM 8797	WT strain	Bauld and Staley, 1976
*G. maris* DSM 8797 DV005	*bla*’::pDV002	This work
*Blastopirellula marina* DSM 3645	WT strain	Schlesner et al., 2004
*B. marina* DSM 3645 DV009	*bla*’::pDV017	This work
*Planctopirus limnophila* DSM3776	WT strain	Hirsch and Müller, 1985
*P. limnophila* DSM3776 DV004	*bla*’::pDV004	This work
*P. limnophila* DSM3776 DV007	Δ*bla*	This work


### Plasmid Description

The oligonucleotides and plasmids used in this work are summarized in **Tables 1** and **2**, respectively. All DNA manipulations were made using standard protocols (Sambrook et al., 2001). Plasmid pMPO1012 was used as template for one-event homologous recombination insertion mutants. It is a ColE1- mobilizable plasmid, which bears the *mut3a-green fluorescent protein (gfp)* gene encoding for a gfp (Cormack et al., 1996) expressed under the *E. coli rrnb* promoter, and an *nptII* kanamycin resistance gene. In order to support homologous recombination, a 1000–1300 bp fragment of the corresponding *bla* genes for each strain was amplified using genomic DNA as a template, and were cloned into the pMPO1012 vector as a *Hin*dIII – directed fragment. If kanamycin was not suitable for selection of the recombinant strains, the tetracycline resistance gene from pUTminiTn5Tc (Herrero et al., 1990) was cloned into the *Sma*I restriction site. Plasmids used for gene deletion in a double event of homologous recombination were derived from pEX18Tc vector (Hoang et al., 1998), which is a suicide plasmid containing *sacB* gene from *Bacillus subtilis* used as counter-selectable marker, and a tetracycline resistance gene. To construct knockout plasmids for *bla* gene, fragment *bla*-up containing 1000–1500 bp sequences upstream of *bla* was amplified by PCR from genomic DNA of the appropriate strain using the primer pairs listed in **Supplementary Table S1**. The fragment *bla-*down containing 1000–1500 bp sequences downstream of *bla* was amplified by PCR using primer cited in **Supplementary Table S1**. The *Eco*RI/*Bam*HI(*Bcl*I)-digested *bla*-up fragment and *Bam*HI(*Bcl*I)/*Hin*dIII(*Sma*I)-digested *bla*-down fragment were then cloned into *Eco*RI/*Hin*dIII(*Sma*I)-digested pEX18Tc by three-way ligation. Finally, the kanamycin resistance gene from the pUTminiTn5km plasmid (Herrero et al., 1990) was subsequently cloned as a *Bam*HI (or *Bcl*I) fragment between the two flanking regions.

**Table 2:** Plasmid used in this work.

Plasmid name	Main features	Source
pMPO1012	Mobilizable, ColE1, Km^R^. Modified from pFPV25 (Valdivia and Falkow, 1996)	Lab collection. Beatriz Mesa, personal communication
pRK2013	Helper plasmid. ColE1. Tra^+^, Km^r^	Figurski and Helinski, 1979
pBF1	Natural marine isolated conjugative plasmid, Hg^r^	Dahlberg et al., 1998
pDV002	1090 bp fragment in pMPO1012, bearing a *bla* gene from *G. maris*. Km^r^	This work
pDV003	1078 bp fragment in pMPO1012, bearing a *bla* gene from *G. obscuriglobus*. Km^r^	This work
pDV004	1081 bp fragment in pMPO1012, bearing a *bla* gene from *P. limnophila*. Km^r^	This work
pDV012	1410 bp upstream and 1243 bp donwstream of *bla* gene from *G. obscuriglobus* flanking a kanamycin resistance gene cloned into pEX18Tc. Km^r^, Tc^r^	This work
pDV013	1472 bp upstream and 1304 bp donwstream of *bla* gene from *G. maris* flanking a kanamycin resistance gene cloned into pEX18Tc. Km^r^, Tc^r^	This work
pDV014	1498 bp upstream and 1443 bp donwstream of *bla* gene from *P. limnophila* flanking a kanamycin resistance gene cloned into pEX18Tc. Km^r^, Tc^r^	This work
pDV017	1234 bp fragment in pMPO1012, bearing a *bla* gene from *B. marina*. Km^r^,Tc^r^	This work
pDV018	pMPO1012 with a tetracycline resistance cloned	This work


### Genetic Modification: Triparental Mating

Genetic modification of *G. obscuriglobus*, *G. maris*, *B. marina*, and *P. limnophila* was performed by triparental mating using 200 μl of an exponentially growing culture (A_600_ ∼0.4) of the donor (see **Table 2**) and helper (pRK2013/*E. coli* DH5α) strains in LB, with the cell pellet of a 15–20 ml culture of the receptor strain (A_600_ ∼0.4). Each culture was previously individually washed in phosphate buffer and resuspended in a total volume of 100 μl that was spotted onto the corresponding agar plates containing cycloheximide. The conjugation patches were incubated for 24 h at 28°C, and resuspended in 1 ml of phosphate buffer. Transconjugants and viable cells were plated with the appropriate antibiotics. Colonies appeared after 12–16 days of incubation at 28°C. Transconjugants were refreshed onto new selection plates with the appropriate antibiotics before they were used to inoculate liquid media. For double recombination events, the pEX18Tc derived- plasmid candidates containing the insertion of the whole plasmid (see **Table 2**) were grown in liquid cultures containing cycloheximide. After 10 days of growth, candidates were segregated onto selective plates containing kanamycin and different concentration of sucrose (0.1, 0.5, 1, 5, and 10%) to counterselect the second recombination event. Transconjugants were verified by PCR and Southern Blotting analysis.

### Genetic Modification: Electroporation

Genetic transformation of *P. limnophila* was performed also by electroporation as described before (Jogler et al., 2011) with some modifications. Fresh electrocompetent cells were prepared from 400 ml of a culture at an A_600_ of 0.4 in modified PYGV. The cells were washed twice with 100 and 50 ml of ice-cold double distilled sterile water and once with 2 ml of ice-cold 10% glycerol. Then, the pellet was resuspended in 400 μl of ice-cold 10% glycerol, and aliquots of 100 μl were dispensed into 0.1-mm gapped electroporation cuvettes along with ∼1 μg of circular DNA (see **Table 2**) and 1 μl of Type-One restriction inhibitor (Epicenter). Electroporation was performed with a Bio-Rad Micropulser (Ec3 pulse, voltage [V] 3.0 kV). Electroporated cells were immediately recovered in 1 ml of cold modified PYGV and incubated at 28°C for 1.5–2 h with shaking. The cells were then plated onto modified PYGV plates supplemented with kanamycin 50 μg ml^-1^ and were incubated at 28°C until colony formation after 5 to 7 days. Colonies were segregated onto fresh selection plates and genotyped by PCR and Southern Blotting analysis.

### Characterisation of Modified Strains by PCR and Southern Blotting

Strains harboring the mutation were genotyped by PCR analysis after fluorescent detection under the microscope. Candidates were checked by amplifying them with paired oligonucleotides located upstream and downstream of the disrupted genes in the genomic DNA (**Supplementary Table S1**). Once candidates were tested by PCR, Southern Blotting analysis with 2 μg of genomic DNA was performed. Genomic DNA was extracted using Wizard Genomic DNA Purification Kit (Promega) with a previous lysozyme treatment (0.5 mg/ml, 1 h at 37°C). The digested DNA was resolved by agarose gel electrophoresis and the DNA transfer was performed as described previously (Brown, 2001). PCR amplicons used as probes were synthetized with the same primers used for genomic amplification for DNA cloning (**Supplementary Table S1**). Probes were labeled according to the manufacturer’s instructions (DIG DNA Labeling Kit, Roche). DIG-labeled probes were detected with anti-Digoxigenin-AP, Fab fragments (Roche), and CSPD (Roche). Visualizations were performed using Chemidoc XRS, and images were analysed with ImageLab 5.0 software.

### Fluorescence Microscopy

For confocal microscopy, cells were grown to an A_600_ ∼0.4, and washed twice with phosphate buffer. After washing, microscopy was performed on a Leica TCS SP5 II microscope using a 63× immersion objective. The fluorescence parameters were fixed for all the strains used in this work. The images were processed by Fiji (version 2.0.0-rc-43/1.50e).

## Results

### Development of a Gene Transfer System and Directed Mutagenesis

#### Gemmata obscuriglobus

In the first place, the sensitivity of *G. obscuriglobus* to kanamycin was evaluated. Saturated cultures of *G. obscuriglobus* were spotted onto LB NaCl-free agar plates supplemented with different concentrations of the antibiotic (5, 10, 20, and 30 μg ml^-1^). After incubation at 28°C for 8–12 days, kanamycin showed growth inhibition at 5 μg ml^-1^ (data not shown), and a concentration of 30 μg ml^-1^ was further used for selection of *G. obscuriglobus* transconjugants.

**FIGURE 2:**
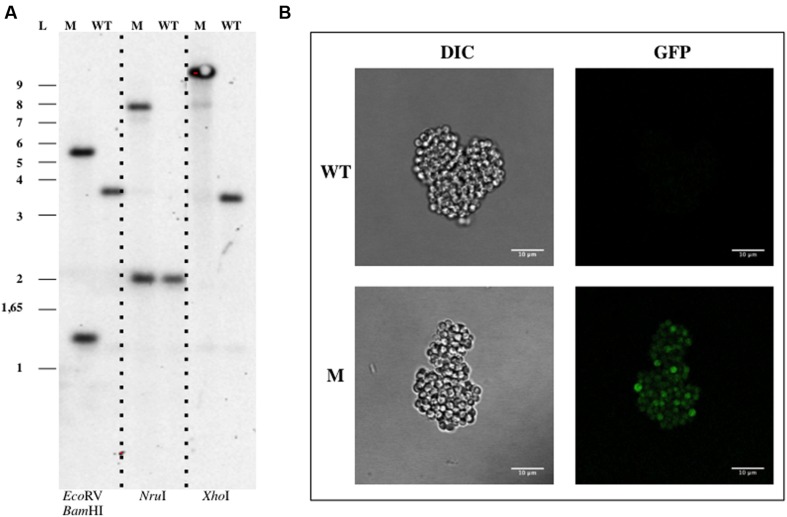
**Genetic manipulation of *Gemmata obscuriglobus* UQM 2246.** **(A)** Southern Blotting analysis of *G. obscuriglobus* DV001. **(B)** Confocal imaging of *G. obscuriglobus*. Abbreviations: WT, wild type; M, mutant; L, ladder; DIC, Differential Interference Contrast.

The pDV003 plasmid, containing a 1078 bp DNA fragment of *bla* gene from *G. obscuriglobus* genome (genbank identifier:163804324:4223-6571) cloned into the pMPO1012 vector, was conjugated into the receptor cells by triparental mating. After 12–16 days of incubation at 28°C isolated colonies were detected in the kanamycin selective medium. No colonies were observed on the selective plates in the mixtures without DNA or when transformed with the empty vector (pMPO1012). The transconjugants (DV001 candidates), which resulted from the integration of a plasmid into the chromosome due to a single event of homologous recombination, were tested by PCR and Southern Blotting analysis using genomic DNA. Southern Blotting assays confirmed the expected pattern of bands sizes after digestion with the appropriate restriction enzymes for the wild type (WT) strains and the insertion mutant (**Figure 2A**). The integration frequency of pDV003 was around 10^-7^–10^-8^ cfu per recipient. In addition, the DV001 transconjugants showed higher fluorescence at the fluorescence microscope as they expressed the gfp encoded in the vector, in comparison with the auto-fluorescent signal of the WT cells (**Figure 2B**).

#### Gimesia maris

The natural plasmid pBF1, which was isolated from marine bacterial communities, was tested for mating in *G. maris*. This plasmid had previously been reported to be transferred from *P. putida* to *G. maris* (Dahlberg et al., 1998). In this work only the transmission of this plasmid was tested, not its stability. To determine if, we could use this plasmid, or its elements, to develop a suitable vector to work with it in *G. maris*, a deeper study of this plasmid was performed. In the first place, the susceptibility of *G. maris* to mercury, the resistance marker of pBF1, was tested. Saturated cultures of the WT strain were spotted onto Maris broth agar plates supplemented with growing concentrations of mercury from a range of 5 to 160 μg ml^-1^. After incubation at 28°C for 10–15 days, *G. maris* showed complete growth inhibition with the lowest mercury concentration tested (data not shown). Biparental mating using pBF1 as donor plasmid were performed as previously described (Dahlberg et al., 1998). Once the cells were conjugated and plated onto Maris broth containing mercury 5 μg ml^-1^, no colony was observed on the selective plates. This data suggest that even though pBF1 vector can be transmitted to *G. maris* by mating, it may not be stable, or the resistance markers are not properly expressed in this strain.

**FIGURE 3:**
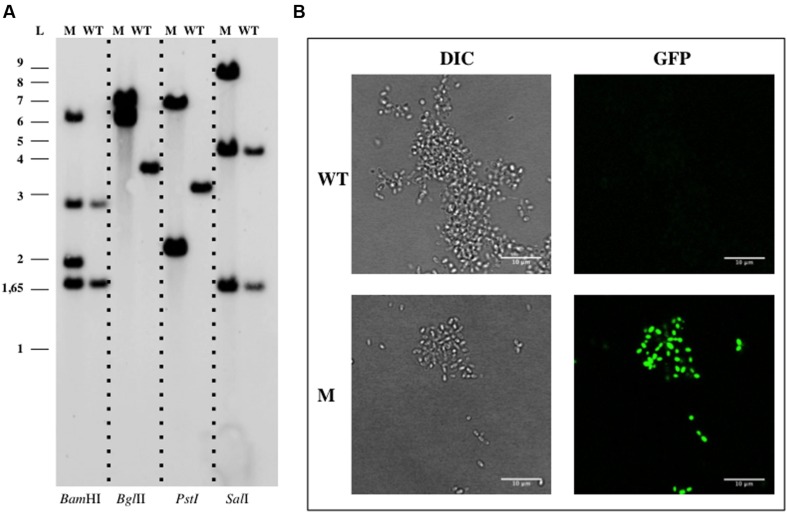
**Genetic manipulation of *Gimesia maris* DSM 8797.** **(A)** Southern Blotting analysis of *G. maris* DV005. **(B)** Confocal imaging of *G. maris*. Abbreviations: WT, wild type; M, mutant; L, ladder; DIC, Differential Interference Contrast.

The construction of an insertion mutant in *G. maris* by a single step of homologous recombination was also assessed. The sensitivity assays for kanamycin resistance were performed by spotting saturated cultures of the WT *G. maris* onto Maris broth agar plates supplemented with several concentrations of this antibiotic (5, 10, 20, 30, 40, 50, and 60 μg ml^-1^). Kanamycin showed a partial growth inhibition at a concentration of 50 μg ml^-1^, so 60 μg ml^-1^ was used for marker selection. For triparental matings in *G. maris*, the plasmid pDV002 containing 1090 bp of the *bla* gene (genbank identifier:149173661:342452-343846) cloned into the pMPO1012 vector, was used as donor. Kanamycin resistant transconjugants, named DV005, grew in Maris broth after incubation at 28°C for 10–15 days. Southern Blotting assays using genomic DNA of the DV005 transconjugants and the WT strains, showed the correct genomic organization as a result of one event of homologous recombination (**Figure 3A**). *G. maris* also displayed increased fluorescence compared to the auto-fluorescence signal, showing that the heterologous *gfp* gene encoded in the plasmid (**Figure 3B**) was expressed in *G. maris*.

#### Blastopirellula marina

Since, *B. marina* is naturally resistant to kanamycin, tetracycline was used as a selection marker (Cayrou et al., 2010). pDV017 plasmid is equivalent to the vectors used for mutagenesis of *G. obscuriglobus*, *P. limnophila*, or *G. maris* but it contains 1234 bp of the *bla* gene from *B. marina* genome (genbank identifier:211606473:c1147370-1146660) and a tetracycline resistance gene.

**FIGURE 4:**
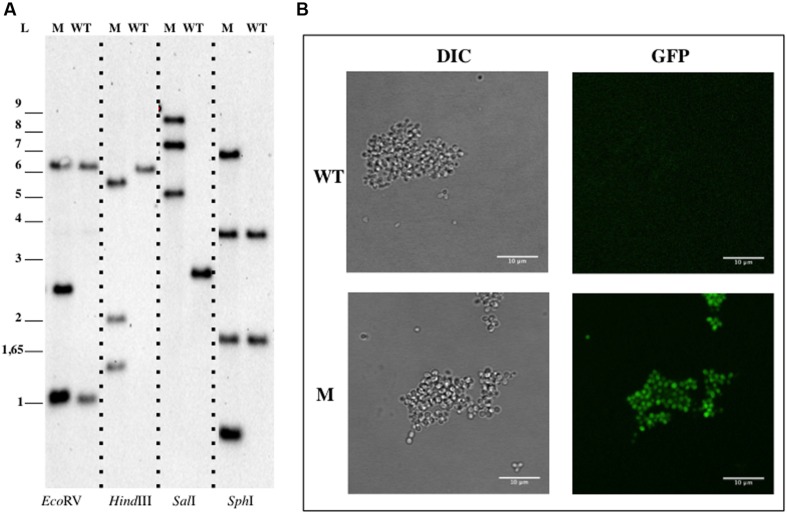
**Genetic manipulation of *Blastopirellula marina* DSM 3645.** **(A)** Southern Blotting analysis of *B. pirellula* DV009. **(B)** Confocal imaging of *B. marina*. Abbreviations: WT, wild type; M, mutant; L, ladder; DIC, Differential Interference Contrast.

pDV017 plasmid was mated into *B. marina* for a single homologous recombination event. After incubation at 28°C for 12–16 days, isolated tetracycline resistant colonies appeared with a frequency of approximately 10^-9^ per recipient (DV009 candidates). No single colonies were obtained in the negative control, however, colonies were detected when the empty plasmid (pDV018) was mated. DV009 transconjugant candidates were tested by PCR analysis, which confirmed the insertion of the plasmid into the chromosome, while Southern Blotting analysis (**Figure 4A**) showed that pDV017 was replicative in *B. marina* and also integrated in the genome by homologous recombination of the *bla* gene at the same time. This was in concordance with the results obtained with the empty plasmid, which was successfully isolated from the transconjugant strains confirming that the pMPO1012- derived plasmids were replicative in *B. marina*. When checked by fluorescence microscopy the mutant strain was also fluorescent as a result of the *gfp* expression (**Figure 4B**).

#### Planctopirus limnophila

**FIGURE 5:**
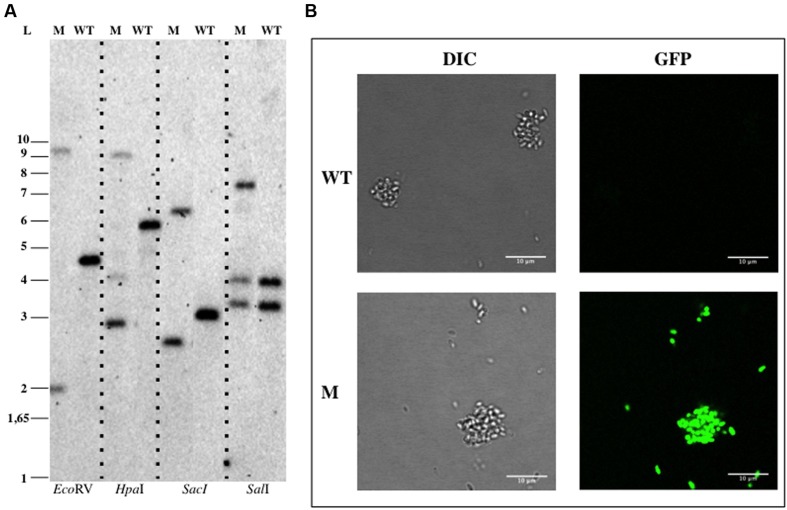
**Genetic manipulation of *Planctopirus limnophila* DSM 3776.** **(A)** Southern Blotting analysis of *P. limnophila* DV004. **(B)** Confocal imaging of *B. marina*. Abbreviations: WT, wild type; M, mutant; L, ladder; DIC, Differential Interference Contrast.

For genetic manipulation of *P. limnophila*, electroporation and conjugation were tested. The vector pDV004 containing 1081 bp of the *bla* gene (genbank identifier: 296120274:807838-809091) was cloned into pMPO1012 vector to perform an insertion mutant by single homologous recombination. Modified strains obtained by electroporation or mating (DV004 candidates) were selected using kanamycin, as previously described (Jogler et al., 2011). No colonies were observed in the negative controls in the mixtures without DNA or with the empty plasmid (pMPO1012). Plasmid integration was verified by PCR using genomic DNA of the DV004 candidates as template and, finally, their genomic arrangement were confirmed by Southern Blotting assays (**Figure 5A**). The integration frequency of the mating was estimated as approximately 10^-6^–10^-7^ cfu per recipient. The *gfp* reporter gene harbored in pDV004 was expressed in *P. limnophila* and non-endogenous fluorescence was clearly detected by fluorescence microscopy (**Figure 5B**).

**FIGURE 6:**
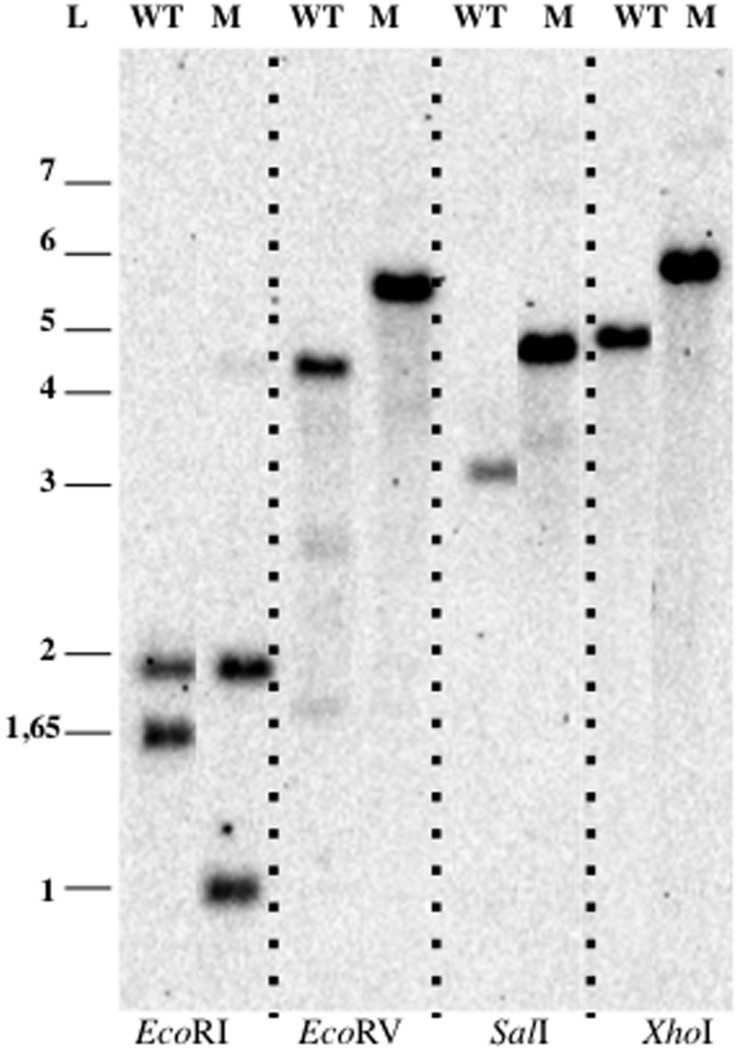
**Genetic manipulation of *P. limnophila* DSM 3776.** Blotting analysis of *P. limnophila* DV007. Abbreviations: WT, wild type; M, mutant; L, ladder.

In order to delete the target gene, cells were electroporated with pDV014, designed for double homologous recombination that contained the flanking regions of the *bla* gene, and plated in Km 50 μg ml^-1^ or Km 50 μg ml^-1^ Tc 5 μg ml^-1^ dishes. No colonies were observed in the plates containing kanamycin plus tetracycline, although colonies appeared in plates with just kanamycin with an estimated frequency of 2x10^-5^ cfu ug^-1^ DNA. These candidates were verified by Southern Blotting analysis (**Figure 6**), and in all cases, a restriction pattern of a *bla* deletion mutant after a double event of homologous recombination was observed. The second recombination event resulted directly without the necessity to force it as described previously (Erbilgin et al., 2014). The tetracycline marker was not conferring resistance to the antibiotic in *P. limnophila*, explaining why no candidates were observed when plated on kanamycin and tetracycline.

## Discussion

Genetic modification combined with phenotypic study of mutants is a powerful tool for the study of gene function. Genetic tools for many bacterial species are available, however, genetic manipulation of non-model organisms, such as Planctomycetes, are frequently hampered by the lack of them. In this work, we describe a protocol for the construction of insertion mutants in *B. marina*, *G. obscuriglobus, G. maris*, and *P. limnophila* by a single event of homologous recombination. In addition, we further show the production of a deletion mutant in *P. limnophila* by a double event of recombination.

In order to construct insertion mutants, we use a ColE1-derived plasmid (pMPO1012), which encodes an antibiotic resistance gene (kanamycin or/and tetracycline) and the gene coding for the gfp. pMPO1012 is a high copy number plasmid in *E. coli*, which yields high amounts of plasmidic DNA from small cultures. Moreover it can be transferred to the receptor cells by conjugation because it contains the origin of conjugal DNA transfer (*oriT*). Surprisingly, we found that the pMPO1012-derived plasmid is replicative in *B. marina*, even though ColE1 replicon has been described as a narrow broad range plasmid, for its replication requires proteins from its host bacterium *E. coli* (Kues and Stahl, 1989). In our case, even though the plasmid is replicative, it is also able to integrate into the bacterial chromosome by homologous recombination of the cloned sequence. Nevertheless, in order to promote a single integration event, a non-replicative plasmid would be more suitable. On the other hand, the description of a replicative plasmid in the Planctomycetes opens up additional possibilities in the genetic manipulation of these organisms.

The *gfp* gene encoded by the pMPO1012-derived plasmids, which is under the control of the *E. coli rrnb* promoter, is successfully expressed in all the strains assayed. The fluorescence of the insertion mutant strains is clearly higher than the one observed in the four WT strains. The *gfp* expression enables rapid identification of clones, originating from a single homologous recombination event, by fluorescence microscopy visualization or fluorescence-assisted cell sorting (FACS). The *gfp* expression is higher in *G. maris* and *P. limnophila* than in *G. obscuriglobus* and *B. marina*. A possible explanation for these differences is that the promoter sequence for the *E. coli* vegetative sigma factor could be more similar to the one of *G. maris* and *P. limnophila*, it is also possible that the codon usage is affecting the different patterns of expression. Thus, heterologous promoters can be functional in Planctomycetes and could be used to express products of interest inside these cells. Depending on the target strain, the promoter and the gene sequence could be optimized to maximize expression.

Although it has been possible to construct a deletion mutant by a double homologous recombination event in *P. limnophila* using pDV014 vector, the construction of deletion mutant in the other three species tested is limited by the number of available resistance gene cassettes and intrinsic resistance of target organisms (Cayrou et al., 2010). An attempt to promote a double homologous recombination event was also performed using plasmid pDV012 and pDV013 that contained the two flanking regions of *bla* gene of *G. obscuriglobus* and *G. maris*, respectively. Those strains were shown to be very sensitive to low concentrations of sucrose (0.1%) and this sugar can thus not be used to counterselect for the *sacB* gene marker in the pEX18Tc- derived plasmid. Also, the tetracycline resistance of the plasmid was not useful in those strains since the first- recombination event candidates were not able to grow on the lowest concentration of tetracycline tested (2.5 μg ml^-1^). Those strains are known to be sensitive to tetracycline (Cayrou et al., 2010), however, it seems that the tetracycline resistance gene cloned into pDV012, pDV013 and also into pDV014, is not expressed or its product is not active in either *G. obscuriglobus, G. maris*, or *P. limnophila* since they are not able to grow in the presence of this antibiotic even when bearing the resistance gene. Therefore, the search of deletion mutants could not be checked by the loss of growth in the presence of tetracycline. Even in *B. marina*, where the tetracycline marker allowed the mutant selection, this antibiotic is required in a very low concentration. New selective markers to combine with kanamycin in *G. maris*, *P. limnophila*, and *G. obscuriglobus* or with tetracycline *in B. marina* are needed to further develop the genetic tools applicable to Planctomycetes.

During the growth of a first cross-over mutant, second cross-over rarely occurs, resulting in a tedious manual screening through colony PCR. Thus, in order to promote a second event of homologous recombination, and excision of the integrated plasmid, a counterselection method would be convenient. In the Planctomycetes, the *sacB* selection method based on sucrose sensitivity (Hoang et al., 1998) is not suitable because sucrose is highly toxic for these organisms, other systems based on conditionally lethal genes, such as *pheS* (Kast, 1994) could be adapted. There are alternative methods to construct deletion mutant, such as the I-SceI endonuclease, the site-specific recombination *cre*/*loxP*-based systems- (Suzuki et al., 2005; Yu et al., 2008; Horzempa et al., 2010; Siegl et al., 2010), or the recent CRISPR (Clustered Regularly Interspaced Short Palindromic Repeats)-associated Cas9 endonucleases system genome-editing tools (Jiang et al., 2013). Nevertheless, they may have other limitations, such as the specificity or the requirements for additional components that should be provided in *trans*, which makes mandatory the finding of replicative plasmids in these organisms.

In this work, we presented the initial tools for genetic manipulation of several Planctomycetes strains, allowing the design of more complex genetic events. This opens up a new field in the characterization of features of these peculiar organisms that will magnify their relevance in the understanding of genome evolution.

## Conclusion

We successfully developed a simple genetic modification approach by triparental mating for four genera of Planctomycetes. This work shows a targeted gene insertion method using gfp for identification and a resistance marker as selection. This approach could be applied to other Planctomycetes strains in order to expand the knowledge of this unusual phylum, broadening the applicability of genetic manipulation in these bacteria, and raising the possibility to manipulate microorganisms of other phyla in the PVC superphylum.

## Author Contributions

ER-M designed and performed the experiments, analyzed the results and wrote the paper. IC and ES contributed to the discussion of the results, and DD designed the general strategy, supervised the work, analyzed data and wrote the paper. All authors reviewed the manuscript.

## Conflict of Interest Statement

The authors declare that the research was conducted in the absence of any commercial or financial relationships that could be construed as a potential conflict of interest.

## Footnotes

1 http://www.dsmz.de
