# Supplementary material for: Development of Genetic Tools for the Manipulation of the Planctomycetes
Source: Front Microbiol. 2016 Jun 16;7:914. doi: 10.3389/fmicb.2016.00914 (PMC4910669; doi:10.3389/fmicb.2016.00914)
Supplement: Supplementary file 1 [file Table_1.PDF]

## *Supplementary Material*

# Genetic tools for the Planctomycetes phylum

Elena Rivas-Marín[1], Inés Canosa[2], Eduardo Santero [2] and Damien P. Devos[1]*

[1] Laboratory of Evolutionary Innovations, Centro Andaluz de Biología del Desarrollo, CSIC, Universidad Pablo de Olavide, Seville, Spain.

[2] Microbiology Area, Centro Andaluz de Biología del Desarrollo, CSIC, Universidad Pablo de Olavide, Seville, Spain.

***Correspondence:** Damien P. Devos, Centro Andaluz de Biología del Desarrollo, CSIC, Universidad Pablo de Olavide, Carretera de Utrera, Km. 1, 41013 Seville, Spain.

damienpdevos@gmail.com

**Supplementary Table 1.** Oligonucleotides used in this work.

| **Primer name** | **Sequence 5´ → 3´** | **Notes** |
|---|---|---|
| Int Gemmata fwd | GGTAAAGCTTGTCCTTGTAGTCGAAGTGC (*Hin*dIII) | To amplify *bla* gene in *G. obscuriglobus.* |
| Int Gemmata rv | CTTGAAGCTTGACCTGTCCGAGTCCTTC (*Hin*dIII) | To amplify *bla* gene in *G. obscuriglobus.* |
| Int Gemmata 2 fwd | TCAGCGCGTCAGCTCCTC | To check DV001 mutant. |
| Int Gemmata 2 rv | GCATGGTGGCGCCATTTAC | To check DV001 mutant. |
| Left Gemmata bla fwd | GGTAGAATTCGACGAGCGCTTATCGGGCGAC (*Eco*RI) | To amplify upstream region of *bla* gene in *G. obscuriglobus.* |
| Left Gemmata bla rv | CTTGGGATCCTGTGCTCCAATCAAAAGGCGG (*Bam*HI) | To amplify upstream region of *bla* gene in *G. obscuriglobus.* |
| Right Gemmata bla 3 fwd | CTTGGGATCCCCATTTACGGTTTCGCGACC (*Bam*HI) | To amplify downstream region of *bla* gene in *G. obscuriglobus.* |
| Right Gemmata bla 3 rv | GGTAAAGCTTCTCCTCATACACCGTGAACC (*Hin*dIII) | To amplify downstream region of *bla* gene in *G. obscuriglobus.* |

| | | |
|---|---|---|
| Int Maris fwd | GGTAAAGCTTACTGATCTCTGATTAATGCAG (*Hin*dIII) | To amplify *bla* gene in *G. maris.* |
| Int Maris rv | CTTGAAGCTTTTCGGCCACAGCGGATATC (*Hin*dIII) | To amplify *bla* gene in *G. maris.* |
| Int Maris 2 fwd | CATGATACTGGGGGATGATG | To check DV005 mutant. |
| Int Maris 2 rv | GTGATCCATATGACCATGCG | To check DV005 mutant. |
| Left Maris bla fwd | GGTAGAATTCGACTGTTATATGCAGGCGTG (*Eco*RI) | To amplify upstream region of *bla* gene in *G. maris.* |
| Left Maris bla rv | CTTGTGATCAATGGCTTCATTTCCAGATCAAG (*Bcl*I) | To amplify upstream region of *bla* gene in *G. maris.* |
| Right Maris bla fwd | GGTATGATCAGAAGTGTAGTACTATAGATGAC (*Bcl*I) | To amplify downstream region of *bla* gene in *G. maris.* |
| Right Maris bla rv | CTTGCCCGGGGTAGTACCGCGAATCGTGAC (*Sma*I) | To amplify downstream region of *bla* gene in *G. maris.* |
| Int Blasto fwd | GGTAAAGCTTCGACCACAACCCCATCCAC (*Hin*dIII) | To amplify *bla* gene in *B. marina.* |
| Int Blasto rv | CTTGAAGCTTCCTGAAGTCCTTGCCAAACG (*Hin*dIII) | To amplify *bla* gene in *B. marina.* |
| Int Blasto 2 fwd | GGCCGAGTTGCATTGATTG | To check DV009 mutant. |
| Int Blasto 2 rv | CGGCAGGCGGCGAAGGATC | To check DV009 mutant. |
| Int Limnophila fwd | GGTAAAGCTTTCCCCTCATTATATCATCAAG (*Hin*dIII) | To amplify *bla* gene in *P. limnophila.* |
| Int Limnophila rv | CTTGAAGCTTTATCGCTGGAATTAGCAATCC (*Hin*dIII) | To amplify *bla* gene in *P. limnophila.* |
| Int Limnophila 2 fwd | GTGGAATGTGCACAGACTG | To check DV004 mutant. |
| Int Limnophila 2 rv | TCATTTGGGCAAGAGGGTC | To check DV004 mutant. |
| Left Limno bla fwd | GGTAGAATTCCCGGGCTGGTTTGCCCATG (*Eco*RI) | To amplify upstream region of *bla* gene in *P. limnophila.* |

| | | |
|---|---|---|
| Left Limno bla rv | CTTGGGATCCGGTCCACCAGAAGCTGTTTC (*Bam*HI) | To amplify upstream region of *bla* gene in *P. limnophila.* |
| Right Limno bla fwd | GGTAGGATCCGCTCTTCCGTGTTCATGCAC (*Bam*HI) | To amplify downstream region of *bla* gene in *P. limnophila.* |
| Right Limno bla rv | CTTGAAGCTTCTCAGGTTCGAGCTGAAGTG (*Hin*dIII) | To amplify downstream region of *bla* gene in *P. limnophila.* |

Underlined nucleotides indicate restriction enzyme sites.
